# A tryptophan residue in the caffeine-binding site of the ryanodine receptor regulates Ca^2+^ sensitivity

**DOI:** 10.1038/s42003-018-0103-x

**Published:** 2018-07-23

**Authors:** Takashi Murayama, Haruo Ogawa, Nagomi Kurebayashi, Seiko Ohno, Minoru Horie, Takashi Sakurai

**Affiliations:** 1 Department of Cellular and Molecular Pharmacology, Juntendo University Graduate School of Medicine, Tokyo, 113-8421 Japan; 2 Institute for Quantitative Biosciences, The University of Tokyo, Tokyo, 113-0032 Japan; 3 Department of Cardiovascular Medicine, Shiga University of Medical Science, Otsu, Shiga 520-2192 Japan; 4 Present Address: Department of Bioscience and Genetics, National Cerebral and Cardiovascular Center, Suita, Osaka 565-8565 Japan

## Abstract

Ryanodine receptors (RyRs) are Ca^2+^ release channels in the sarcoplasmic reticulum of skeletal and cardiac muscles and are essential for muscle contraction. Mutations in genes encoding RyRs cause various muscle and arrhythmogenic heart diseases. Although RyR channels are activated by Ca^2+^, the actual mechanism of Ca^2+^ binding remains largely unknown. Here, we report the molecular basis of Ca^2+^ binding to RyRs for channel activation and discuss its implications in disease states. RyR1 and RyR2 carrying mutations in putative Ca^2+^ and caffeine-binding sites were functionally analysed. The results were interpreted with respect to recent near-atomic resolution RyR1 structures in various ligand states. We demonstrate that a tryptophan residue in the caffeine-binding site controls the structure of the Ca^2+^-binding site to regulate the Ca^2+^ sensitivity. Our results reveal the initial step of RyR channel activation by Ca^2+^ and explain the molecular mechanism of Ca^2+^ sensitization by caffeine and disease-causing mutations.

## Abstract (web summary)

Takashi Murayama et al. report the molecular basis of calcium binding to ryanodine receptors, a process essential for muscle contraction. They find that a tryptophan residue in the caffeine binding site controls the structure of the calcium binding site, affecting calcium sensitivity.

## Introduction

Ryanodine receptors (RyRs) are Ca^2+^ release channels in the sarcoplasmic reticulum of skeletal and cardiac muscles that play a pivotal role in excitation-contraction coupling^1,2^. RyRs are huge (~2 MDa) homotetrameric protein complexes with a large cytoplasmic structure and a cation channel domain of six transmembrane segments at the carboxyl (C)-terminus^3,4^. Type 1 RyR (RyR1) is a skeletal muscle isoform and mutations in *RYR1* cause several muscle diseases, including malignant hyperthermia and central core disease^5,6^. Type 2 RyR (RyR2) is expressed mainly in the heart, and mutations in *RYR2* are associated with various arrhythmogenic heart diseases, including catecholaminergic polymorphic ventricular tachycardia (CPVT)^7,8^. To date, a total of nearly 800 disease-related mutations have been identified in *RYR1* and *RYR2* genes.

The RyR channel mediates Ca^2+^-induced Ca^2+^ release (CICR), in which the channel is directly activated by Ca^2+^^2,9^. The binding of Ca^2+^ to the Ca^2+^-binding site is an initial step for channel activation^9^; therefore, regulation of the Ca^2+^-binding is important for channel activity and is implicated in disease states. Indeed, CICR activity is enhanced by many *RYR1* mutations found in malignant hyperthermia and central core disease^10–12^ and by *RYR2* mutations identified in CPVT^13–15^. The molecular details of the Ca^2+^ binding site and its regulation, however, remain largely unknown.

**Fig. 1:**
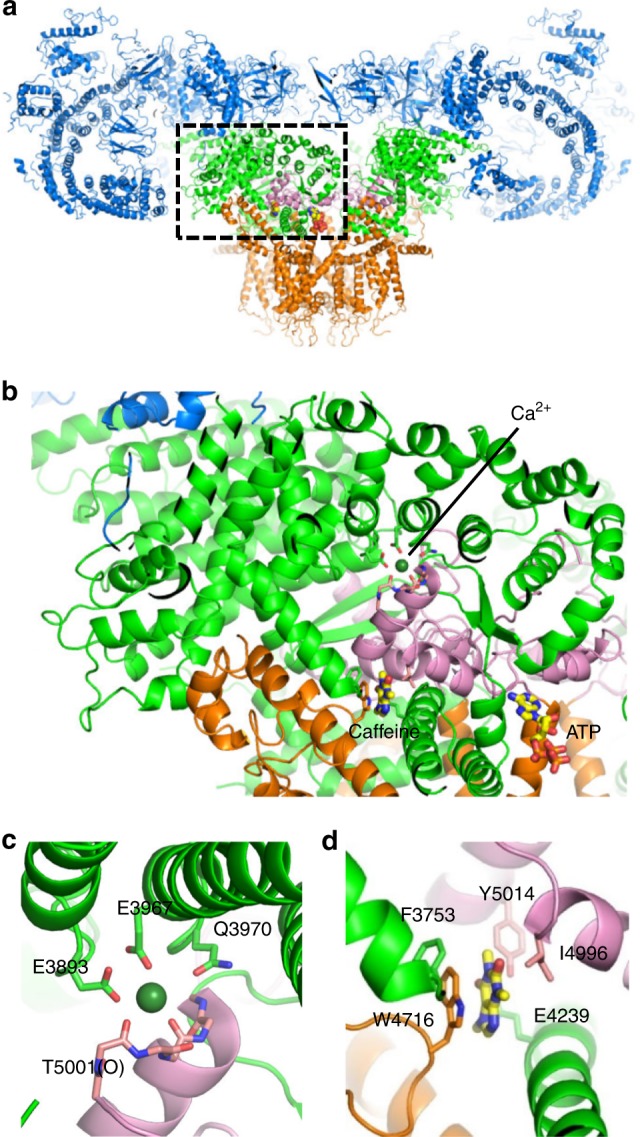
Putative Ca^2+^-binding and caffeine-binding sites in RyR1. **a** The architecture of RyR1 is depicted in ribbon representation, with the shell (residues 1-3666) colored *blue*, the core solenoid (CSol, residues 3667-4253) *green*, the transmembrane region and S6c (residues 4540-4956) *orange*, and the C-terminal domain (CTD, residues 4957-5037) *pink*. Atomic coordinates (PDB accession code: 5T9V) from des Georges et al.^20^ were used. **b** Closed-up view of boxed area in (**a**). The binding sites of Ca^2+^ **c** and caffeine **d**, with proposed interacting residues labeled are depicted in ribbon representation

Recent cryo-electron microscopy structures of RyR1 and RyR2 have revealed a complex architecture, involving a superhelical scaffold in the cytoplasmic domain and a channel domain of the voltage-gated ion channel superfamily^16–20^. Furthermore, des Georges et al.^20^ reported putative binding sites for three major activating ligands, Ca^2+^, ATP, and caffeine, on a near-atomic resolution structure of RyR1. They proposed that the Ca^2+^-binding site is located in the core domain just above the transmembrane domain (Fig. 1a, b) and consists of several negatively charged residues in the core solenoid (CSol) domain and carboxyl-terminal domain (CTD) (Fig. 1c). Caffeine, a xanthine derivative, is a potent and common activator of all known RyR isoforms and greatly enhances Ca^2+^ sensitivity of the channel^21,22^. The putative caffeine-binding site is located just below the Ca^2+^-binding site and consists of several hydrophobic residues from different domains (Fig. 1d). The close proximity of Ca^2+^- and caffeine-binding sites suggests direct links between the two sites in regulating Ca^2+^ sensitivity. Indeed, a missense mutation (W4645R) of a tryptophan in the putative caffeine-binding site of human RyR2 has been reported to cause CPVT^23^, indicating the physiological significance of the caffeine-binding site.

To elucidate the molecular basis of Ca^2+^ binding for channel activation and to assess the role of this mechanism in disease states, we conducted functional analysis of RyR1 and RyR2 channels carrying mutations in these sites using a heterologous expression system in HEK293 cells. Based on high similarity in ligand-binding sites between RyR1 and RyR2, the results were interpreted with respect to recently reported near-atomic resolution structures of RyR1. We identified these as the actual binding sites and demonstrate that a tryptophan residue in the caffeine-binding site controls the structure of the Ca^2+^-binding site to regulate the Ca^2+^ sensitivity. Our results reveal the initial step of RyR channel activation by Ca^2+^ and explain the molecular mechanism of Ca^2+^ sensitization by caffeine and disease-causing mutations.

## Results

### Tryptophan affects caffeine response and Ca^2+^ sensitivity

As a first step to address the molecular mechanism of Ca^2+^ binding to RyRs and its regulation via the caffeine-binding site, we analysed a CPVT mutation in the putative caffeine-binding site of human RyR2 (W4645R)^23^. The corresponding mutation (W4644R, see Supplementary Table 1) was introduced in mouse RyR2, and the mutant channel was heterologously expressed in HEK293 cells, which do not express an endogenous RyR. An alanine-substituted mutant (W4644A) was also generated for comparison. Expression of the mutant RyR2 was confirmed by Western blotting (Supplementary Figure 1).

**Fig. 2:**
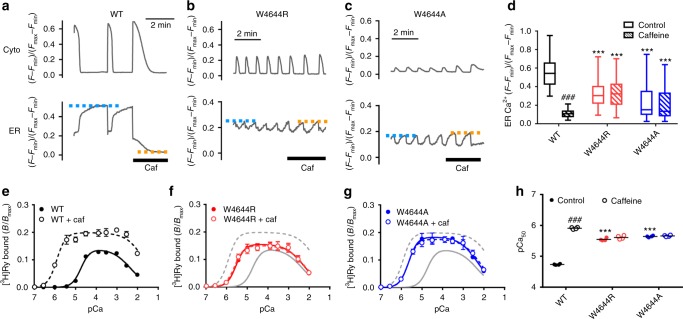
Functional analysis of tryptophan mutations in the putative caffeine-binding site in RyR2. **a**–**c** Representative traces of [Ca^2+^]_i_ (*Cyto*) and [Ca^2+^]_ER_ (*ER*) signals of HEK293 cells expressing WT RyR2 (**a**), W4644R (**b**) and W4644A (**c**) G-GECO1.1 and R-CEPIA1er were co-transfected to monitor [Ca^2+^]_i_ and [Ca^2+^]_ER_, respectively. Measurements were carried out 4–6 h after induction of RyR2, where ER Ca^2+^ was still retained. [Ca^2+^] is expressed as (*F − F*_min_)/(*F*_max_*− F*_min_), in which *F*_min_ and *F*_max_ were determined in the presence of ionomycin with BAPTA and CaCl_2_, respectively. Caffeine (10 mM) was applied at the time indicated by the thick bar. Blue and orange dotted lines indicate upper levels of [Ca^2+^]_ER_ in control and caffeine-containing Krebs solution, respectively. **d** Upper level of [Ca^2+^]_ER_ in WT (*n* = 60), W4644R (*n* = 49), and W4644A (*n* = 63) RyR2 cells in control (*open columns*) and with 10 mM caffeine (*hatched columns*). Data are given as box and whisker plots displaying the minimum, first quartile, median, third quartile, and maximum values. The mutants exhibited more frequent Ca^2+^ oscillations with reduced [Ca^2+^]_ER_ than WT and did not respond to caffeine. *** Indicates statistical significance with *p* < 0.0001 vs WT using one-way ANOVA followed by Dunnett’s post hoc test. ###*p* < 0.0001 vs control using unpaired two-tailed Student’s *t*-test. **e**–**g** Ca^2+^-dependent [^3^H]ryanodine binding of WT (**e**), W4644R (**f**) and W4644A (**g**), with (*open circles*) or without (*closed circles*) 10 mM caffeine. Data are given as mean ± SEM (*n* = 4). Data were fitted to Eqs. (1–3) for biphasic Ca^2+^ dependence using fixed Hill coefficients of 2.0 and 1.0 for activating and inactivating Ca^2+^ sites, respectively, for WT and all the mutants. The mutants exhibited enhanced sensitivity for activating Ca^2+^ compared with WT in the absence of caffeine. They did not respond to caffeine. Open and closed circles are mostly overlapped for the mutants. **h** Summary of pCa values for half-maximum activation (pCa_50_) of WT, W4644R, and W4644A with (*hatched columns*) or without (*open columns*) 10 mM caffeine. Data are given as mean (*horizontal bar*) and individual values (*circles*). *** Indicates statistical significance with *p* < 0.0001 vs WT using one-way ANOVA followed by Dunnett’s post hoc test. ###*p* < 0.0001 vs control using unpaired two-tailed Student’s *t*-test

The phenotype of the mutant RyR2 channel was initially investigated by monitoring intracellular Ca^2+^ homeostasis of HEK293 cells using G-GECO1.1 and R-CEPIA1er to simultaneously detect Ca^2+^ in the cytoplasm ([Ca^2+^]_i_) and ER lumen ([Ca^2+^]_ER_), respectively^15^ (Fig. 2a–c). [Ca^2+^] is expressed as (*F − F*_min_)/(*F*_max_*− F*_min_), in which *F*_min_ and *F*_max_ were determined in the presence of ionomycin with 1,2-bis(*o*-aminophenoxy)ethane-*N*,*N*,*N’*,*N’*-tetraacetic acid (BAPTA) plus cyclopiazonic acid and CaCl_2_, respectively (Supplementary Figure 2). In cells expressing WT RyR2, spontaneous Ca^2+^ oscillations occurred with concomitant decrease in [Ca^2+^]_ER_, indicating Ca^2+^ release from ER via the RyR2 channels (Fig. 2a). Cells expressing W4644R and W4644A RyR2 showed more frequent Ca^2+^ oscillations with reduced [Ca^2+^]_ER_ compared to the WT RyR2 cells (Fig. 2b–d). Tetracaine, a known RyR inhibitor, effectively suppressed Ca^2+^ oscillations and greatly increased [Ca^2+^]_ER_ of the mutant RyR2 cells (Supplementary Figure 3). These features have been shown to be typical gain-of-function phenotype of CPVT mutants^15,24^. In the WT RyR2 cells, caffeine triggered massive Ca^2+^ release from the ER (Fig. 2a). However, it did not trigger Ca^2+^ release or affect Ca^2+^ oscillations of the mutant RyR2 cells, suggesting that these mutants are unresponsive to caffeine (Fig. 2b–d).

Next, we determined Ca^2+^-dependent [^3^H]ryanodine binding to microsomes isolated from HEK293 cells. Since ryanodine specifically binds to the open RyR channel, amount of bound [^3^H]ryanodine quantitatively monitors the channel activity^21,22^. WT RyR2 exhibited biphasic Ca^2+^ dependence (Fig. 2e). W4644R and W4644A showed biphasic Ca^2+^ dependence with an enhanced Ca^2+^ sensitivity for activation (Fig. 2f, g). pCa value for 50% activation (pCa_50_) was greater in the mutants than in WT RyR2 (Fig. 2h). Again, W4644R and W4644A did not respond to caffeine, which greatly sensitized WT RyR2 to activation by Ca^2+^. Taken together, these results suggest that the tryptophan residue is essential for the action of caffeine. In addition, and more interestingly, the tryptophan is also involved in the physiological regulation of Ca^2+^ sensitivity.

To address whether the tryptophan residue is also important for the other RyR isoforms, we generated HEK293 cells that express RyR1 carrying alanine substitution of W4716 in the putative caffeine-binding site and performed functional analysis by Ca^2+^ imaging. Caffeine caused Ca^2+^ transients of WT RyR1 cells in a dose-dependent manner with an EC_50_ of ~1 mM^11,12^ (Supplementary Figure 4). W4716A RyR1 cells did not exhibit caffeine-induced Ca^2+^ transients, but they still responded to 4-CmC, another activator of the RyR1 channel^25^. These findings indicate an important role of the tryptophan residue in caffeine action in RyR1.

### Tryptophan-mediated regulation of Ca^2+^ sensitivity

**Fig. 3:**
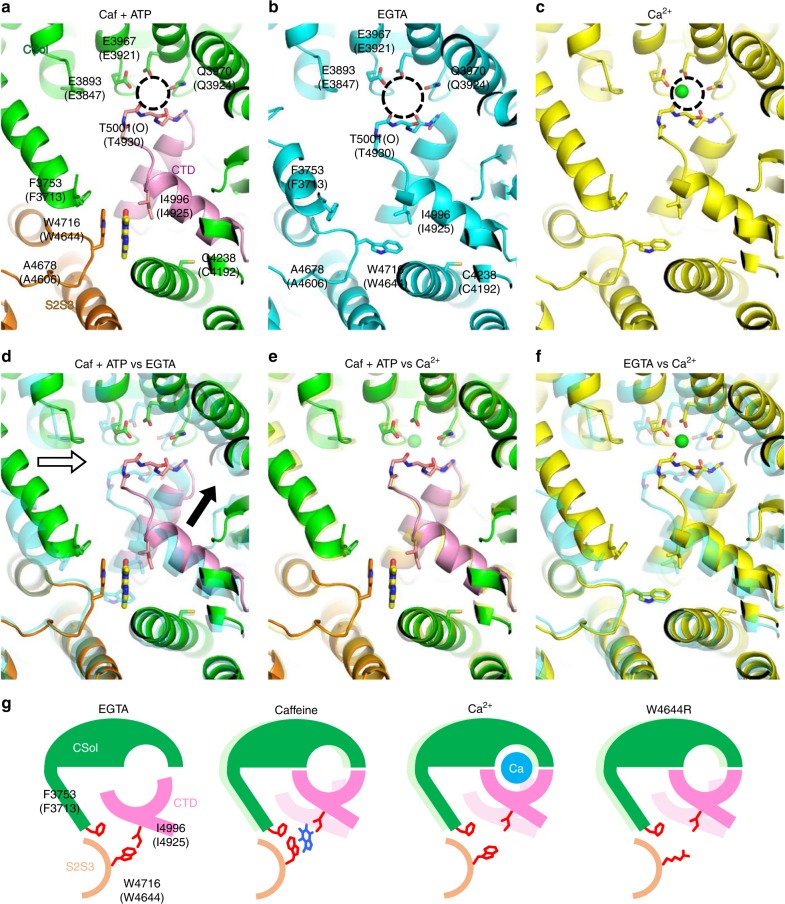
Conformational changes of Ca^2+^-binding and caffeine-binding sites in the presence and absence of ligands. **a**–**c** The architecture of putative Ca^2+^-binding and caffeine-binding sites under different conditions. Structures in the presence of (**a**) caffeine and ATP (Caf + ATP) (PDB accession code: 5TAP), (**b**) EGTA (5TB0) and (**c**) Ca^2+^ (5T15) are depicted in ribbon representation. The Ca^2+^-binding pocket is indicated by a dotted circle. In Caf + ATP, the core solenoid (CSol) domain, S2S3 domain, and CTD are colored in *green*, *orange,* and *pink*, respectively. The likely interacting residues of the putative Ca^2+^-binding and caffeine-binding sites of rabbit RyR1 and mouse RyR2 (in parentheses) are labeled. **d**–**f** Structures in two different conditions are overlaid: (**d**) Caf + ATP and EGTA, (**e**) Caf + ATP and Ca^2+^, and (f) EGTA and Ca^2+^. **d** In Caf + ATP, CSol moves a ~2 Å rightward (*white arrow*) and CTD moves a ~2 Å upward (*black arrow*) compared with in EGTA. **e** Conformational changes by Caf + ATP closely resemble those by Ca^2+^. **f** Similar movements of CSol and CTD were observed by Ca^2+^. **g** Hypothetical conformational changes of Ca^2+^-binding and caffeine-binding sites in RyR2 by interaction between tryptophan and isoleucine. In EGTA, W4644, and I4925 interact to pull the CTD toward the S2S3 domain, which makes the Ca^2+^-binding pocket larger and less favorable for Ca^2+^. Caffeine breaks the interaction by rotating the tryptophan side chain (Caf + ATP). This moves the CTD toward the CSol to make the Ca^2+^-binding pocket smaller and more favorable for Ca^2+^. Similar conformational changes occur in response to Ca^2+^. Mutation in tryptophan (W4644R) may also break the interaction to cause an upward shift of the CTD, resulting in enhanced Ca^2+^ sensitivity. *Light colors* in caffeine, Ca^2+^, W4644R indicate locations of the CSol and CTD in the EGTA state

The putative binding sites for Ca^2+^ and caffeine are located close to each other (see Fig. 1); therefore, it is reasonable to expect that the caffeine-binding site directly regulates the Ca^2+^-binding site. To address the conformational changes caused by caffeine, it is necessary to compare the structure of RyR2 with or without caffeine. The structure of RyR2 with caffeine has not yet been solved and so we used RyR1 structures instead. The structures included RyR1 in the presence of caffeine plus ATP (Caf + ATP, PDB accession number 5TAP) (Fig. 3a), in the absence of Ca^2+^ (EGTA, 5TB0) (Fig. 3b), and in the presence of Ca^2+^ (5T15) (Fig. 3c). Comparison of the Caf + ATP and EGTA structures revealed that orientation of the tryptophan indole group (W4716 in RyR1 and W4644 in RyR2) is dynamically changed by the binding of caffeine (Fig. 3d). In addition, the size of the Ca^2+^-binding pocket is smaller in the Caf + ATP structure (the area demarked by the dotted circle in Fig. 3b is ~38 Å^2^, while the equivalent area shown in Fig. 3a is ~24 Å^2^), which is brought about by a ~2 Å upward shift of the CTD (Fig. 3d, black arrow) and a ~2 Å rightward shift of the CSol (Fig. 3d, white arrow). Interestingly, the overall structure around the Ca^2+^-binding site in Caf + ATP closely resembles that in the Ca^2+^ bound structure (Fig. 3e). Moreover, the size of the Ca^2+^-binding pocket in the two states is similar (the dotted circle shown in Fig. 3c is ~22 Å^2^). Thus, the Ca^2+^-binding site in the presence of caffeine appears more favorable for the binding of Ca^2+^ compared with the site in the absence of caffeine. This conformational change reasonably explains enhancement of Ca^2+^ sensitivity by caffeine.

By looking at the binding site in more detail, we found that isoleucine (I4996 in RyR1 and I4925 in RyR2) in the CTD is located close to the tryptophan in the EGTA state (Fig. 3b). Based on the finding, we hypothesized that an interaction between the tryptophan and the isoleucine may stabilize the CTD toward S2S3 (Fig. 3g). This may make the Ca^2+^-binding pocket larger and less favorable for the binding of Ca^2+^. The binding of caffeine will alter the orientation of the tryptophan indole group, which breaks the interaction causing movement of the CTD upward to make the Ca^2+^-binding pocket smaller and more favorable for Ca^2+^ binding. This reasonably explains why the CPVT mutant (W4644R) enhances the Ca^2+^ sensitivity; the mutated residues may weaken the interaction to move the CTD upward.

The amino acid residues around the putative Ca^2+^- and caffeine-binding sites are well conserved in three RyR isoforms (Supplementary Figure 5). We therefore compared structures of the putative Ca^2+^- and caffeine-binding sites of pig RyR2 in the presence of Ca^2+^ and in its absence (with EGTA) using available PDB data^19^ (Supplementary Figure 6). The overall structures and conformational changes by Ca^2+^ resembled those of RyR1: (1) the Ca^2+^-binding pocket was considerably smaller in the presence of Ca^2+^ compared with EGTA, (the area demarked by the dotted circle in Supplementary Figure 6 is ~34 Å^2^ in EGTA, while the equivalent area shown in the presence of Ca^2+^ is ~18 Å^2^), (2) the CTD moved upward in the presence of Ca^2+^ (~3 Å), (3) the CSol moved to the right in the presence of Ca^2+^ (~3 Å), and (4) hydrophobic residues responsible for caffeine action (tryptophan, isoleucine, and phenylalanine) are all conserved in RyR2.

### Negatively charged and polar residues in the Ca^2+^-binding site

**Fig. 4:**
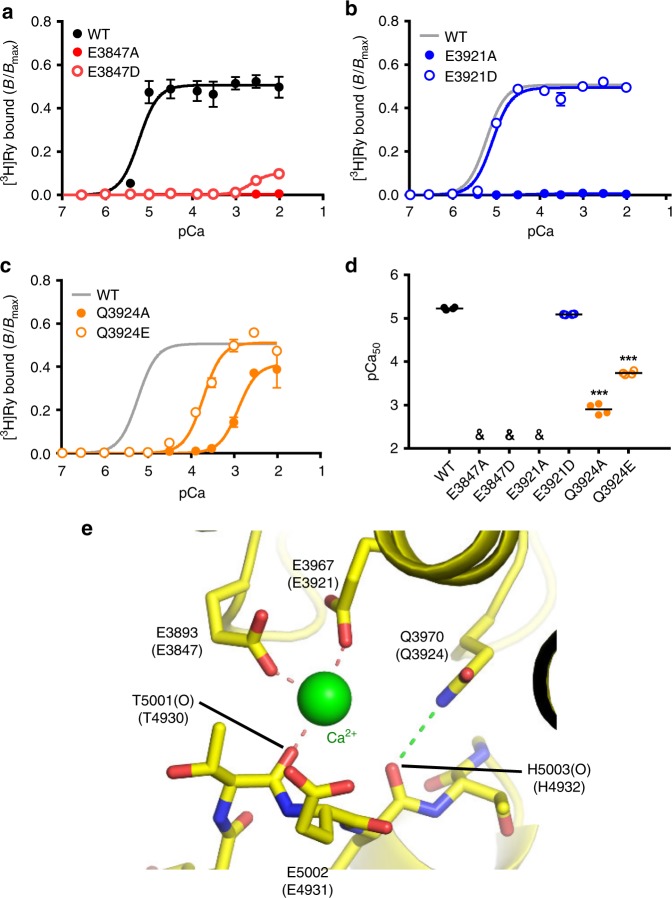
Mutations at negatively charged and polar residues affect Ca^2+^ coordination in the putative Ca^2+^-binding site. **a**–**c** Ca^2+^-dependent [^3^H]ryanodine binding of RyR2 carrying mutations in the putative Ca^2+^-binding site; E3847 (**a**), E3921 (**b**) and Q3924 (**c**). Assays were carried out in high salt medium without Mg^2+^ to remove inactivation via the low-affinity Ca^2+^/Mg^2+^ site. Data are given as mean ± SEM (*n* = 4). **d** Summary of pCa_50_ values of WT and the mutants. Data are given as mean (*horizontal bar*) and individual values (*circles*). &, not determined. ***p < 0.0001 vs WT using one-way ANOVA followed by Dunnett’s post hoc test. Mutations differentially reduced or lost Ca^2+^ sensitivity for activation. **e** The architecture of putative Ca^2+^-binding site (5T15). The possible coordination of oxygen atoms to Ca^2+^ are indicated by orange dashed lines, and possible hydrogen bond stabilizing the coordination geometry by green dashed lines

To verify the above hypothesis, we at first analysed the putative Ca^2+^-binding site, in which Ca^2+^ is proposed to be coordinated directly by E3893, E3967, and the carbonyl oxygen of T5001, and also indirectly by Q3970^20^ in RyR1 (Fig. 1). Mutations at the coordinated residues are expected to reduce affinity for Ca^2+^; therefore, Ca^2+^-dependent activation should shift toward higher Ca^2+^ concentrations. Thus, RyR2s carrying mutations in the corresponding residues were tested using a Ca^2+^-dependent [^3^H]ryanodine binding assay. For an accurate measurement of Ca^2+^ dependent activation, assays were carried out in the presence of high salt (1 M NaCl) and no Mg^2+^, which removes inactivation by low-affinity Ca^2+^/Mg^2+^-binding sites^21,22^. Under these conditions, WT RyR2 exhibited monophasic Ca^2+^ dependence with pCa_50_ of ~5 (Fig. 4a). Alanine substitution caused total loss of binding in E3847 and E3921, and greatly reduced the Ca^2+^ sensitivity in Q3924 (Fig. 4a–d). These functional results are consistent with the structural data, strongly supporting the proposed structure of the Ca^2+^-binding site. Interestingly, aspartate substitution caused different phenotypes in the two glutamates; E3847D showed minimal binding at higher Ca^2+^ concentrations (Fig. 4a), while E3921D exhibited similar Ca^2+^ sensitivity as the WT (Fig. 4b). In addition, glutamate substitution of the glutamine (Q3924E), the corresponding mutation in human RyR2 (Q3925E) being associated with arrhythmogenic diseases^26^, also reduced Ca^2+^ sensitivity (Fig. 4c). This suggests that the amide group of Q3924 has an important role in regulation of the Ca^2+^ sensitivity. Based on the structure, we propose that the amide group of Q3924 may form a hydrogen bond with the carbonyl oxygen of H4932 in the CTD which stabilizes the CSol-CTD interface (Fig. 4e).

### Tryptophan and isoleucine negatively regulates Ca^2+^ sensitivity

**Fig. 5:**
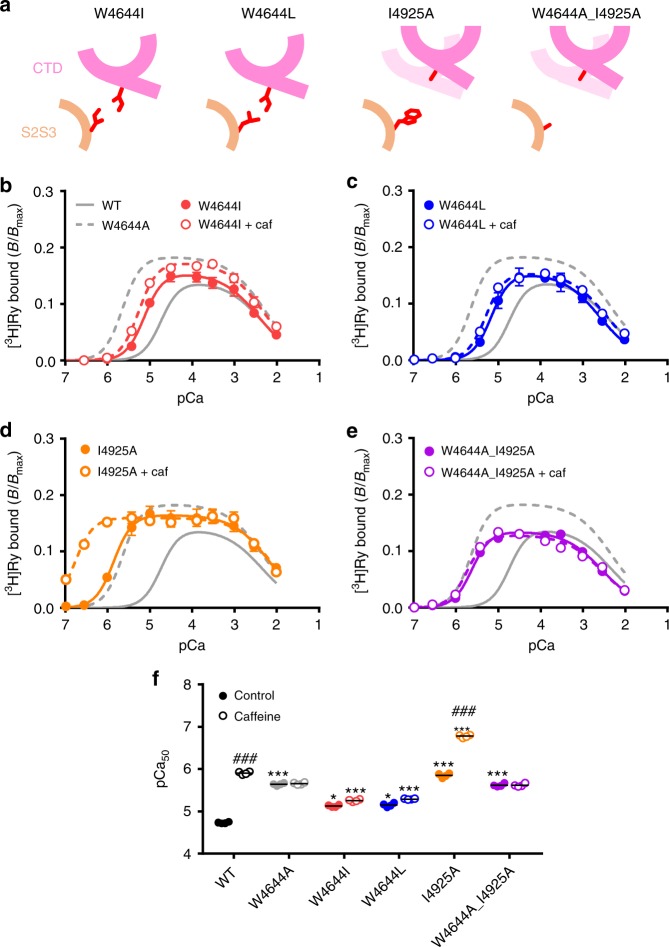
Verification of interaction between tryptophan and isoleucine in the putative caffeine-binding site. **a** Schematic diagrams of four mutants at W4644 and/or I4925 with expected interactions and movement of the CTD. *Light pink* in I4925A and W4644A_I4925A indicates location of the CTD in WT. **b**–**e** Ca^2+^-dependent [^3^H]ryanodine binding to W4644I (**b**), W4644L (**c**), I4925A (**d**) and I4925A_W46444A (**e**) in the presence (*closed circles*) and absence (*open circles*) of 10 mM caffeine. Data are given as mean ± SEM (*n* = 4). **f** Summary of pCa_50_ values of WT and the mutants with (*hatched columns*) or without (*open columns*) 10 mM caffeine. Data are given as mean (*horizontal bar*) and individual values (*circles*). * and *** indicate statistical significance with *p* = 0.004 and *p* < 0.0001, respectively, vs WT using one-way ANOVA followed by Dunnett’s post hoc test. ### indicates *p* < 0.0001 vs control using unpaired two-tailed Student’s *t*-test

We next verified our hypothesis in the putative caffeine-binding site. We generated two mutants, W4644I and W4644L. Since side chains of isoleucine and leucine are longer and more hydrophobic than that of alanine, it is expected that interaction with the isoleucine is partially preserved in these mutants (Fig. 5a). Consistent with the hypothesis, Ca^2+^ sensitivity of W4644I and W4644L was considerably lower than that of W4644A (Fig. 5b, c, f), although it was still higher than that of WT. Moreover, W4644I and W4644L did not respond to caffeine (Fig. 5b, c, f), indicating an essential role of the tryptophan residue in caffeine action.

If interaction between tryptophan and isoleucine is essential in the regulation of Ca^2+^ sensitivity, the isoleucine side chain should also have an important role in maintaining the interaction. We therefore mutated the isoleucine to alanine (I4925A). I4925A exhibited greatly enhanced Ca^2+^ sensitivity comparable to that of W4644A (Fig. 5d, f). Interestingly, Ca^2+^ sensitivity was not increased further in the double mutant (W4644A_I4925A) (Fig. 5e, f). These results indicate that the enhanced Ca^2+^ sensitivity in W4644A and I4925A mutants occurs through a common mechanism, and that the maximum effect was caused by just a single alanine substitution. Taken together, these findings strongly support our hypothesis that the interaction between tryptophan and isoleucine is essential in the regulation of Ca^2+^ sensitivity.

### Ca^2+^-sensitizing effect via phenylalanine-tryptophan interaction

The above results appear to reasonably explain the Ca^2+^-sensitizing effect of caffeine by interaction between tryptophan and isoleucine. However, we found that caffeine further sensitized I4925A to Ca^2+^ (Fig. 5d, f). An additional mechanism of Ca^2+^-sensitizing effect of caffeine is necessary to explain this phenomenon, since the interaction is thought to be completely lost in I4925A. In the structures, we noticed that side chain of tryptophan (W4716 in RyR1 and W4644 in RyR2) gets to phenylalanine (F3753 in RyR1 and F3713 in RyR2) within ~2 Å in the presence of Caf + ATP (Fig. 3a). Thus, the rotated indole group of tryptophan is likely to interact with phenylalanine, which may cause a ~2 Å rightward shift of CSol to make the Ca^2+^-binding pocket more suitable for Ca^2+^.

**Fig. 6:**
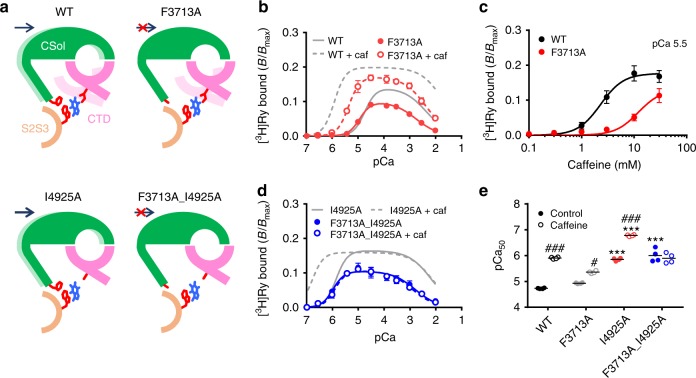
Verification of interaction between tryptophan and phenylalanine in the putative caffeine-binding site. **a** Schematic diagrams of conformation of Ca^2+^-binding and caffeine-binding sites for WT, F3713A, I4925A and F3713A_I4925A in the presence of caffeine with expected interactions and movement of the CSol and CTD. *Light colors* indicate locations of the CSol and CTD in the absence of caffeine. Caffeine shifts the CSol rightward in WT and I4925A via the interaction between tryptophan and phenylalanine, but not in F3713A or F3713A_I4925A because of loss of the phenylalanine residue. **b** Ca^2+^-dependent [^3^H]ryanodine binding to F3713A with (*open circles*) or without (*closed circles*) 10 mM caffeine. The Ca^2+^ sensitization of F3713A by caffeine was about half that of WT. Data are given as mean ± SEM (*n* = 4). **c** Caffeine-dependent activation of [^3^H]ryanodine binding to WT and F3713A at pCa 5.5. F3713A showed lower sensitivity to caffeine than WT. Data are given as mean ± SEM (*n* = 4). **d** Ca^2+^-dependent [^3^H]ryanodine binding to F3713A_I4925A with (*open circles*) or without (*closed circles*) 10 mM caffeine. Data are given as mean ± SEM (*n* = 4). **e** Summary of pCa_50_ values of WT and the mutants with (*hatched columns*) or without (*open columns*) 10 mM caffeine. Data are given as mean (*horizontal bar*) and individual values (*circles*). * and *** indicate statistical significance with *p* < 0.004 and *p* < 0.0001, respectively, vs WT using one-way ANOVA followed by Dunnett’s post hoc test. ###*p* < 0.0001 vs control using unpaired two-tailed Student’s *t*-test

To verify this possibility, we substituted the phenylalanine in RyR2 with alanine (F3713A). Loss of phenylalanine side chain is expected to weaken the interaction, which causes a reduced Ca^2+^ sensitizing effect of caffeine (Fig. 6a). In the absence of caffeine, F3713A exhibited a reduced [^3^H]ryanodine binding with slightly higher Ca^2+^ sensitivity (pCa_50_ = 4.93 ± 0.03) than WT (pCa_50_ = 4.79 ± 0.03) (Fig. 6b, e). Caffeine sensitized the mutant to Ca^2+^, but the effect was rather weak compared with WT (Fig. 6b, c, e). We next tested the effect of the F3713A mutation on I4825A. If the phenylalanine-tryptophan interaction is responsible for an additional Ca^2+^ sensitization mechanism, the double mutant (F3713A_I4925A) should be unresponsive to caffeine (Fig. 6a). The Ca^2+^ sensitivity of F3713A_I4925A was as high as that of I4925A, but was not further sensitized by caffeine at all (Fig. 6d, e). These results support the above hypothesis that the phenylalanine-tryptophan interaction mediates an additional mechanism of caffeine action.

To address whether isoleucine and phenylalanine residues are also important for RyR1, functional analysis was done with HEK293 cells that express RyR1 carrying I4966A or F3753A mutation (Supplementary Figure 4). I4966A showed higher caffeine sensitivity with a reduced amplitude in caffeine-induced Ca^2+^ release, indicating a typical gain-of-function phenotype^11,12^. F3573A was much less responsive to caffeine in releasing Ca^2+^ compared with WT. The fact that 4-CmC caused large Ca^2+^ release in F3753A suggests similar level of ER Ca^2+^ as in WT. A caffeine-sensitive gain-of-function of I4996A and a reduced caffeine sensitivity of F3753A are consistent with the phenotype of I4925A and F3713A in RyR2, respectively.

### Caffeine-binding site is a target for disease-associated mutations

**Fig. 7:**
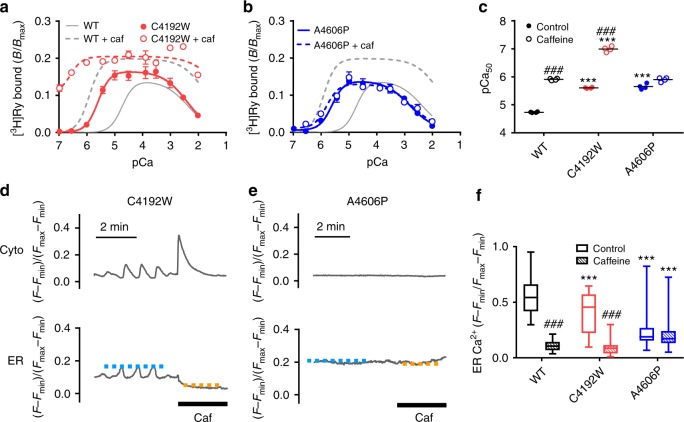
Disease-associated mutations with enhanced Ca^2+^ sensitivity located near the caffeine-binding site. **a**, **b** Ca^2+^-dependent [^3^H]ryanodine binding to C4192W (**a**) and A4606P (**b**) with (*open circles*) or without (*closed circles*) 10 mM caffeine. Data are given as mean ± SEM (*n* = 4). **c** Summary of pCa_50_ values of WT, C4192W and A4606P with (*hatched columns*) or without (*open columns*) 10 mM caffeine. The two mutants exhibited similar enhancement in Ca^2+^ sensitivity but responded differentially to caffeine. Data are given as mean (*horizontal bar*) and individual values (*circles*). ***Indicates statistical significance with *p* < 0.0001 vs WT using one-way ANOVA followed by Dunnett’s post hoc test. ###*p* < 0.0001 vs control using unpaired two-tailed Student’s *t*-test. **d** Representative traces of [Ca^2+^]_i_ (*cyto*) and [Ca^2+^]_ER_ (*ER*) signals of HEK293 cells expressing C4192W and A4606P. Measurements were carried out 4–6 h after induction of RyR2 when ER Ca^2+^ was still retained. Caffeine (10 mM) was applied at the time indicated by the thick bar. C4192W cells showed Ca^2+^ oscillations and responded to caffeine. In contrast, A4606P cells did not show any Ca^2+^ oscillation nor caffeine-induced Ca^2+^ release (*right*). Blue and orange dotted lines indicate upper levels of [Ca^2+^]_ER_ in control and caffeine-containing Krebs solution, respectively. **e** Upper level of [Ca^2+^]_ER_ in C4192W (*n* = 42) and A4606P (*n* = 61) RyR2 cells for control (*open columns*) and 10 mM caffeine treatment (*hatched columns*) compared with WT (*n* = 60). Data for WT are the same with those in Fig. 2d. Data are given as box and whisker plots. ***Indicates statistical significance with *p* < 0.0001 vs WT using one-way ANOVA followed by Dunnett’s post hoc test. ###*p* < 0.0001 vs control using unpaired two-tailed Student’s *t*-test

Many disease-associated mutations have been found around ligand-binding sites in human RyR2^27^. By functional screening of these mutations, we identified two CPVT mutations in human RyR2, C4193W^28^ and A4607P^29^, which greatly enhance the Ca^2+^ sensitivity. C4193W mutation was de novo and a patient suffered from syncope exhibited bidirectional ventricular tachycardia after the exercise stress test (Supplementary Figure 7). In the RyR1 structure, a corresponding cysteine (C4238) is located in the helix of the TaF domain, which is located just beneath the CTD, whereas a corresponding alanine (A4678) exists in the helix of S2S3, which is in close proximity to W4716 (Fig. 3). The two corresponding mutants (C4192W and A4606P) in mouse RyR2 demonstrated eightfold higher Ca^2+^ sensitivity than WT in [^3^H]ryanodine binding (Fig. 7a–c). Based on our calculation with the three parameters for CICR, the channel activity of these mutants was estimated to be more than 60-fold greater than that of WT at resting (100 nM) Ca^2+^ (Supplementary Figure 8), suggesting an arrhythmogenic potential. Interestingly, they exhibited different responses to caffeine: C4192W was further sensitized to Ca^2+^ by caffeine, but no activation was observed in A4606P (Fig. 7a–c). In HEK293 cells, C4192W demonstrated frequent Ca^2+^ oscillations with a reduced [Ca^2+^]_ER_ (Fig. 7d). A4606P cells exhibited a reduced [Ca^2+^]_ER_ but no Ca^2+^ oscillations (Fig. 7e, Supplementary Figure 2). Tetracaine greatly increased [Ca^2+^]_ER_ of these mutant RyR2 cells (Supplementary Figure 3), indicating a gain-of-function phenotype. Again, caffeine caused massive Ca^2+^ release from the ER in C4192W but not in A4606P (Fig. 7d–f).

The phenotypes of these mutations could be explained by molecular mechanisms (Fig. 3). In cysteine-to-tryptophan mutation, steric hindrance may occur between the large indole group of tryptophan and α-helix of the CTD, in which the isoleucine is located. This may disrupt the interaction between tryptophan and isoleucine but may not affect positioning or orientation of tryptophan side chain; therefore, the mutant can still respond to caffeine via the phenylalanine-tryptophan interaction, as shown with isoleucine-to-alanine mutant (see Fig. 5c). In alanine-to-proline mutation, in contrast, incorporation of proline would kink the helix to alter the structure of the S2S3 helix, affecting the positioning or orientation of tryptophan side chain to disable caffeine binding, as observed with tryptophan-substituted mutants (see Fig. 2).

## Discussion

Ca^2+^ binding is the initial step in the activation of RyR channels and Ca^2+^ sensitivity is crucial for channel regulation. However, the molecular mechanism and regulation of the Ca^2+^ binding is poorly understood. In this study, we addressed this question by a combination of phenotype analysis of various RyR mutants, and interpretation by recently reported structures of the putative Ca^2+^- and caffeine-binding sites of RyR1 at near-atomic resolution. We verified the proposed binding sites to be the actual binding sites. Furthermore, we demonstrated that (1) the caffeine-binding site negatively regulates the Ca^2+^ sensitivity through an interaction between tryptophan in S2S3 and isoleucine in CTD, (2) caffeine sensitizes RyR to Ca^2+^ in two ways: by breaking the interaction between the tryptophan and the isoleucine and by forming another interaction between the tryptophan and a phenylalanine in Csol, and (3) CPVT mutations weaken the tryptophan-isoleucine interaction to enhance the Ca^2+^ sensitivity. Thus, the tryptophan residue serves as a switch to regulate the Ca^2+^ sensitivity. Our findings revealed the molecular basis for Ca^2+^ binding to RyR and provide an explanation for disease states.

We performed phenotype analysis of the mutant RyRs using intracellular Ca^2+^ ([Ca^2+^]_i_ and [Ca^2+^]_ER_) measurements and [^3^H]ryanodine binding. These assays measure the activity of the RyR channels^21,22^. Thus, our results by functional analysis provide an indirect evidence for binding of Ca^2+^ or caffeine to their binding sites. However, highly quantitative nature of [^3^H]ryanodine binding enabled us to address the Ca^2+^ sensitivity and the effect of caffeine in the mutant RyRs. We interpreted the functional results of mouse RyR2 with rabbit RyR1 structure, since there is no structural information about the Ca^2+^- and caffeine-binding sites in the reported RyR2 structure^19^. One might concern about misinterpretation by different isoforms. However, we are confident of our strategy for the following reasons. First, the effect of caffeine is common between RyR1 and RyR2^21,22^. Second, the residues responsible for caffeine binding are highly conserved within the three mammalian RyR isoforms (Supplementary Figure 5). Third, structures of Ca^2+^- and caffeine-binding sites and conformational change by Ca^2+^ are common between RyR1 and RyR2 (Supplementary Figure 6). Finally, the proposed mechanism can reasonably explain the phenotypes of RyR1s carrying mutations at hydrophobic residues in the caffeine-binding sites (Supplementary Figure 4). These findings strongly indicate that the strategy used in this study is valid and the conclusions can be commonly applied to both RyR1 and RyR2.

In the putative Ca^2+^-binding site, Ca^2+^ is surrounded by negatively charged residues, including two glutamate residues and one glutamine residue^20^ (Fig. 1). It has been proposed that the glutamate residues are involved in direct coordination of Ca^2+^, whereas the glutamine residue may contribute to indirect Ca^2+^ coordination^20^. We showed that alanine substitution of glutamates completely abolished Ca^2+^-dependent [^3^H]ryanodine binding, whereas that of glutamine greatly reduced but not abolished the Ca^2+^ sensitivity (Fig. 4). These findings strongly support the proposed model of the Ca^2+^-binding site. The functional analysis also provided important information about Ca^2+^-binding sites that has not been predicted. First, aspartate substitution of the two glutamates caused different phenotypes: E3847D caused a severe reduction in Ca^2+^ sensitivity, whereas E3921D exhibited no or only minor change. This implies that the approximate location of these residues against Ca^2+^ is different: E3921 might be more closely located to Ca^2+^ than E3847. Second, Q3924E caused a great reduction of Ca^2+^ sensitivity, indicating an importance of the amide group of the glutamine. Based on the structure, we propose that the amide group of the glutamine may form a hydrogen bond with the carbonyl oxygen of histidine in the CTD to stabilize the CSol-CTD interface (Fig. 4).

The putative caffeine-binding site of RyR1 consists of hydrophobic residues, i.e., tryptophan, isoleucine and phenylalanine (Fig. 1), among which tryptophan has been predicted to be most important for caffeine binding^20^. We demonstrated that mutations at the tryptophan in RyR1 (Supplementary Figure 4) and RyR2 (Fig. 2) caused complete loss of caffeine action. This strongly supports the proposed model of caffeine-binding site. In contrast, caffeine still enhanced the Ca^2+^ sensitivity of the mutant carrying an alanine substitution of isoleucine (Fig. 5) or phenylalanine (Fig. 6). Thus, these residues are not essential for caffeine binding; rather they contribute to Ca^2+^ sensitization by caffeine via interaction with the tryptophan.

Our current working model of regulation of the Ca^2+^-binding site by caffeine-binding site is as follows. In the absence of caffeine, interaction between tryptophan in S2S3 and isoleucine in CTD keeps the lower half of the Ca^2+^-binding site in CTD apart from the upper half in the CSol. This makes the Ca^2+^-binding pocket larger and less favorable for Ca^2+^ binding. Caffeine binds to the caffeine-binding site so as to align in parallel with an indole group of the tryptophan by rotating its side chain. This breaks the tryptophan-isoleucine interaction, leading to upward shift of CTD by a ~2 Å toward the upper half of the Ca^2+^-binding site to make Ca^2+^-binding pocket smaller that is more favorable for Ca^2+^ binding (Fig. 3). In addition, side chain of the tryptophan then interacts with phenylalanine in CSol, which causes rightward shift of the upper half of the Ca^2+^-binding site by a ~2 Å to make the Ca^2+^-binding pocket more suitable for Ca^2+^ binding (Fig. 5). The two mechanisms are independent of each other and cooperatively sensitize the channel to activating Ca^2+^.

The presence of two Ca^2+^ sensitizing mechanisms raises the question of their relative contributions. This could be answered by phenotypes of mutants lacking either interaction. The Ca^2+^ sensitization effect of caffeine on phenylalanine-to-alanine mutant was about half that on WT (Fig. 6). On the other hand, isoleucine-to-alanine mutant exhibited 10-fold greater Ca^2+^ sensitivity than WT in the absence of caffeine, and caffeine further sensitized the mutant to Ca^2+^ by 10-fold (Fig. 5). Taken together, we estimate that the two mechanisms have roughly the same contribute to the Ca^2+^ sensitization by caffeine. Another question is which kind of interactions occurs in the caffeine-binding site. Since tryptophan, isoleucine, and phenylalanine are all hydrophobic residues, hydrophobic interactions by van der Waals force are mostly plausible. Alternatively, steric effect of tryptophan residue is also possible since tryptophan has a bulky side chain. More detailed structures will provide evidence for the underlying mechanism of interactions.

We used the structure of RyR1 with caffeine plus ATP (5TAP), due to no other available structures with caffeine. This might raise a concern that some structure changes in the Ca^2+^-binding and caffeine-binding sites are caused by ATP. However, the putative binding site of ATP is ~25 Å apart from the caffeine-binding site^20^. In addition, it has been shown that ATP does not change the Ca^2+^ sensitivity nor influence the Ca^2+^-sensitizing action of caffeine^21,22^. Thus, it seems unlikely that ATP directly affects the caffeine-binding site, although we cannot completely exclude the possibility.

We demonstrate that conformation of the Ca^2+^-binding site with Caf + ATP (5TAP) is overlapped with that with Ca^2+^ (5T15) (Fig. 3). One possible assumption is that caffeine can mimic the conformational change induced by Ca^2+^. However, functional analysis revealed that Ca^2+^ is essential for activation of the RyR channels even in the presence of caffeine (see Fig. 2). des Georges et al.^20^ demonstrated that conformation with Ca^2+^ or with Caf + ATP is in the primed state, in which the cytoplasmic assembly and activation module are moved but the gate remains closed, and that all the three ligands can induce further conformational changes to open the gate. Indeed, dynamic conformational changes occur in the Ca^2+^-binding and caffeine-binding sites in the structure with Ca^2+^/Caf/ATP (5T9V) (Supplementary Figure 9).

Since the caffeine-binding site negatively regulates the channel by reducing Ca^2+^ sensitivity, it is reasonable to expect that mutations disrupting the caffeine-binding site sensitize the channel to Ca^2+^ to cause disease. Indeed, we demonstrate that a CPVT mutation in tryptophan in the caffeine-binding site (W4645R in human RyR2^23^) greatly sensitizes the RyR2 channel to Ca^2+^ (Fig. 2). We also identified two additional CPVT mutations near the caffeine-binding site in human RyR2, C4193W^28^ and A4607P, that exhibit enhanced Ca^2+^ sensitivity (Fig. 7). Thus, the caffeine-binding site and the surrounding regions are candidates for disease-associated mutations in RyR2. A similar situation may also occur with RyR1, although we could not detect corresponding mutations in RyR1. RyRs exhibit global conformational changes upon channel activation^19,20,30,31^; therefore, it is possible that certain mutations apart from those in the Ca^2+^- and caffeine-binding sites also affect these sites by a long-range allosterism. Comprehensive functional analysis of the mutant channels will provide more information.

What we propose in this study is a very simple and clear-cut mechanism of Ca^2+^ sensitization by the caffeine-binding site. Thus, it is highly possible that the site functions physiologically in vivo. Caffeine is a xanthine derivative (methylxanthine) produced in plants^32^. Although methylxanthines are not present in animals, including humans, xanthine is a metabolite of purine nucleotides in humans. It has been reported that 9-methyl-7-bromoeudistomin D (MBED), an artificial β-carboline analogue, has a potent Ca^2+^ releasing activity by binding to the caffeine-binding site^33^. Some β-carbolines may be formed naturally in humans^34^. Thus, it is quite possible that some derivatives of xanthine or β-carbolines act as the endogenous regulators of RyRs through the caffeine-binding site to regulate physiological Ca^2+^ sensitivity in vivo.

## Methods

### Generation of stable inducible HEK293 cell lines

HEK293 cells, stably and inducibly expressing WT and mutant forms of RyR1^12^ and RyR2^15^ were generated using the Flp-In T-REx system (Life Technologies, CA, USA). Each mutation was introduced into rabbit RyR1 (GenBank accession number X15209.1) or mouse RyR2 (NM_023868.2) cDNA by inverse polymerase chain reaction using primers listed in Supplementary Table 2. The mutated cDNA fragment was subcloned into the expression vector (pcDNA5/FRT/TO). Flp-In T-REx HEK293 cells were maintained in Dulbecco’s modified Eagle’s media supplemented with 10% fetal calf serum and 2 mM L-glutamine. The stable inducible cell lines were generated by co-transfection of the expression vector with the pOG44 vector encoding Flp recombinase according to the manufacturer’s instructions. Transfected cells were re-plated one day after transfection and the growth medium was replaced with selective medium containing 100 µg ml^−1^ hygromycin. The selective medium was changed every three days until the hygromycin-resistant foci were identified. Several hygromycin-resistant foci were tested for the inducible expression of RyRs and clones with suitable expression were selected and used for experiments.

### Single-cell Ca^2+^ imaging

Single-cell Ca^2+^ imaging was carried out in HEK293 cells expressing WT or mutant RyR1 and RyR2^15,35^. In Ca^2+^ measurements with RyR2, the Ca^2+^ signals from cytoplasm ([Ca^2+^]_i_) and ER lumen ([Ca^2+^]_ER_) were monitored using G-GECO1.1 (a gift from Robert Campbell from University of Alberta, Addgene plasmid # 32445)^36^ and R-CEPIA1er (a gift from Masamitsu Iino, The University of Tokyo)^37^, respectively. Cells were transfected with cDNAs for these Ca^2+^ indicators 28–32 h before measurement. Expression of RyR2 was induced by doxycycline (2 µg/ml) 4–6 h before measurement depending on the mutant. Experiments were carried out with HEPES-buffered Krebs solution (140 mM NaCl, 5 mM KCl, 2 mM CaCl_2_, 1 mM MgCl_2_, 11 mM glucose and 5 mM HEPES at pH 7.4). In experiments with RyR1, cells were loaded with fluo-4 AM 24 h after induction and fluorescence signals in response to caffeine (0.03–10 mM) were determined. At the end of each experiment, *F*_min_ and *F*_max_ were obtained with 0Ca Krebs solution containing 20 µM ionomycin, 5 mM BAPTA, and 20 µM cyclopiazonic acid and 20Ca Krebs solution containing 20 µM ionomycin and 20 mM CaCl_2_, respectively^15^. Averaged *F*_min_ was 7.655% of *F*_max_ for R-CEPIA1er and 0% for G-GECO1.1 and fluo-4. The fluorescence signal (*F − F*_min_) was normalized to the maximal fluorescence intensity (*F*_max_*− F*_min_). Measurements were carried out at 26°C.

### Preparation of microsomes

HEK293 cells expressing RyR2 mutants (5 × 150 mm dishes) were collected and rinsed twice with phosphate-buffered saline. The cell pellets were resuspended with 5 ml of 0.3 M sucrose, 20 mM 3-morpholinopropanesulfonic acid (MOPS), pH 7.4 with a protease inhibitor cocktail and processed for nitrogen cavitation for 15 min at 1000 psi. The homogenate was centrifuged at 1000 × g for 5 min and the supernatant was ultracentrifuged for 30 min at 100,000 × g. The microsomal pellet was resuspended with 5 ml of the above buffer and ultracentrifuged again. The pellet was resuspended with 1 ml of the above buffer, quickly frozen with liquid nitrogen, and stored at −80 °C until used.

### [^3^H]Ryanodine binding and parameter analysis

Microsomes isolated from HEK293 cells expressing WT and mutant RyR2 were incubated for 1 h at 25°C with 5 nM [^3^H]ryanodine in a medium containing 0.17 M NaCl, 20 mM 3-(*N*-morpholino)-2-hydroxypropanesulfonic acid (MOPSO) at pH 7.0, 2 mM dithiothreitol, 1 mM AMP, 1 mM MgCl_2_ and various concentrations of free Ca^2+^ buffered with 10 mM ethylene glycol-bis(2-aminoethylether)-N,N,N’,N’-tetraacetic acid (EGTA)^15,35^. Free Ca^2+^ concentrations were calculated using WEBMAXC STANDARD (http://web.stanford.edu/~cpatton/webmaxcS.htm)^38^. The [^3^H]ryanodine binding data (*B*) were normalized to the maximum number of functional channels (*B*_max_), which was separately determined by Scatchard plot analysis using varied concentrations (3–20 nM) of [^3^H]ryanodine in a high-salt medium. The resultant *B*/*B*_max_ represents the averaged activity of each mutant. For mutants regarding the Ca^2+^-binding sites, medium containing 1 M NaCl and no Mg^2+^ was used to remove inactivation through the low-affinity Ca^2+^/Mg^2+^ site.

To determine the parameters of Ca^2+^-dependent activity, the data were fitted to the following equation:

1 $$A = A_{{\mathrm{max}}} \times f_{\mathrm{A}} \times \left( {1 - f_{\mathrm{I}}} \right)$$

where *A* is the binding at the specified Ca^2+^, *A*_max_ is the gain that determines the maximal attainable binding, and *f*_A_ and *f*_I_ are fractions of the activating Ca^2+^ site (A-site) and inactivating Ca^2+^ site (I-site) bound to Ca^2+^, respectively^39^. *f*_A_ and *f*_I_ at the specified Ca^2+^ concentration ([Ca^2+^]) are expressed as:

2 $$f_A = \left[ {{\mathrm{Ca}}^{2 + }} \right]^{\,n{\mathrm{A}}}/\left( {\left[ {{\mathrm{Ca}}^{2 + }} \right]^{\,n{\mathrm{A}}} + K_{\mathrm{A}}^{\,n{\mathrm{A}}}} \right)$$

3 $$f_I = \left[ {{\mathrm{Ca}}^{2 + }} \right]^{\,n{\mathrm{I}}}/\left( {\left[ {{\mathrm{Ca}}^{2 + }} \right]^{\,n{\mathrm{I}}} + K_I^{n{\mathrm{I}}}} \right)$$

where *K*_A_ and *K*_I_ are dissociation constants, and *n*_A_ and *n*_I_ are Hill coefficients for Ca^2+^ of A- and I-sites, respectively. The Hill coefficients were set at 2.0 and 1.0 for *n*_A_ and *n*_I_, respectively, for WT and all the mutant channels, which maximize the sum of *R*^2^ values for curve fitting. Curve fitting was performed using the Prism 6 software (GraphPad Software, La Jolla, CA, USA). The curves for *f*_A_, 1 – *f*_I_, and *A* are shown in Supplementary Figure 6. For estimation of CICR activity at resting [Ca^2+^]_i_, *A* at pCa = 7 was calculated by Eqs. (1–3) using the obtained parameters (*K*_A_, *K*_I_, and *A*_max_).

### Western blotting

Microsomal proteins (10 μg) were separated by sodium dodecyl sulfate polyacrylamide gel electrophoresis with a 3–12% gel and transferred onto a polyvinylidene fluoride membrane. Western blotting was performed using antibodies for pan RyR^40^ (1: 5000 dilution) and calnexin (C4731, Sigma-Aldrich, MO, USA) (1: 5000 dilution).

### Genetic analysis

The patient carrying C4193W mutation in RyR2^28^ was diagnosed with CPVT and introduced to Shiga University of Medical Science for genetic analysis. The patient and her family provided written informed consent in accordance with the guidelines approved by our institutional review boards (23-128-3). Genomic DNA was extracted from peripheral blood lymphocytes by use of the DNA Isolation Kit (Roche Diagnostics GmbH, Mannheim, Germany). Genetic screening for 50 genes related to inherited arrhythmias including *RYR2*, *CASQ2*, *KCNJ2* and *TRDN* were performed by using a bench-top next generation sequencer (NGS) (MiSeq, Illumina, San Diego, CA, USA). Detected variants were confirmed by Sanger methods.

### Data analysis

Statistical analysis was performed using Prism 6 (GraphPad Software, Inc., La Jolla, CA, USA). Unpaired two-tailed Student’s *t*-test and one-way ANOVA followed by Dunnett’s test, were performed to compare two groups and three or more groups, respectively. Structural figures were prepared with PyMOL (The PyMOL Molecular Graphics System, Schrödinger).

### Data availability

The authors declare that the data supporting the findings of this study are available within the paper and its supplementary information files. The PDB accession numbers we used in this paper are 5TAP, 5TB0, 5T15, 5T9V, 5GO9, 5GOA.

## Electronic supplementary material

Supplementary Information
