# Supplementary material for: A tryptophan residue in the caffeine-binding site of the ryanodine receptor regulates Ca2+ sensitivity
Source: Commun Biol. 2018 Jul 23;1:98. doi: 10.1038/s42003-018-0103-x (PMC6123685; doi:10.1038/s42003-018-0103-x)
Supplement: Supplementary file 1 — Supplementary Information [file 42003_2018_103_MOESM1_ESM.pdf]

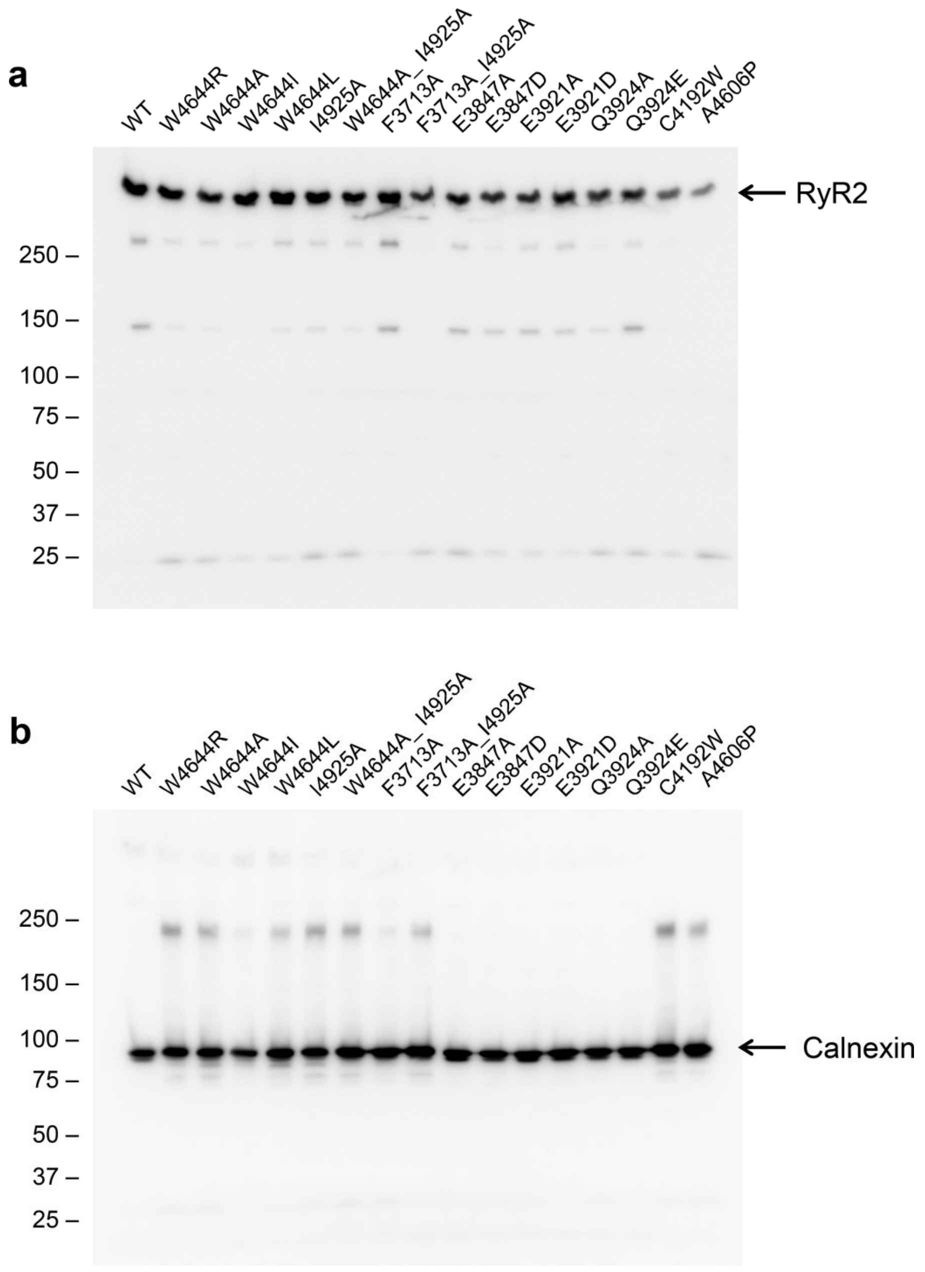

Supplementary Figure 1. Expression of WT and mutant RyR2 channels in HEK293 cells. Western blotting of RyR2 (a) and calnexin (b, loading control) was carried out with microsomes from HEK293 cells expressing WT and disease-associated mutants (W4644R, W4644A, W4644I, W4644L, I4925A, W4644A_I4925A, F3713A, F3713A_I4925A, E3847A, E3847D, E3921A, E3921D, Q3924A, Q3924E, C4192W and A4606P). The mobility of the RyR2 band was similar between WT and the mutants. The intensity was similar in most mutants, but varied in some mutants, e.g., A4606P which showed substantially lower intensity than WT.

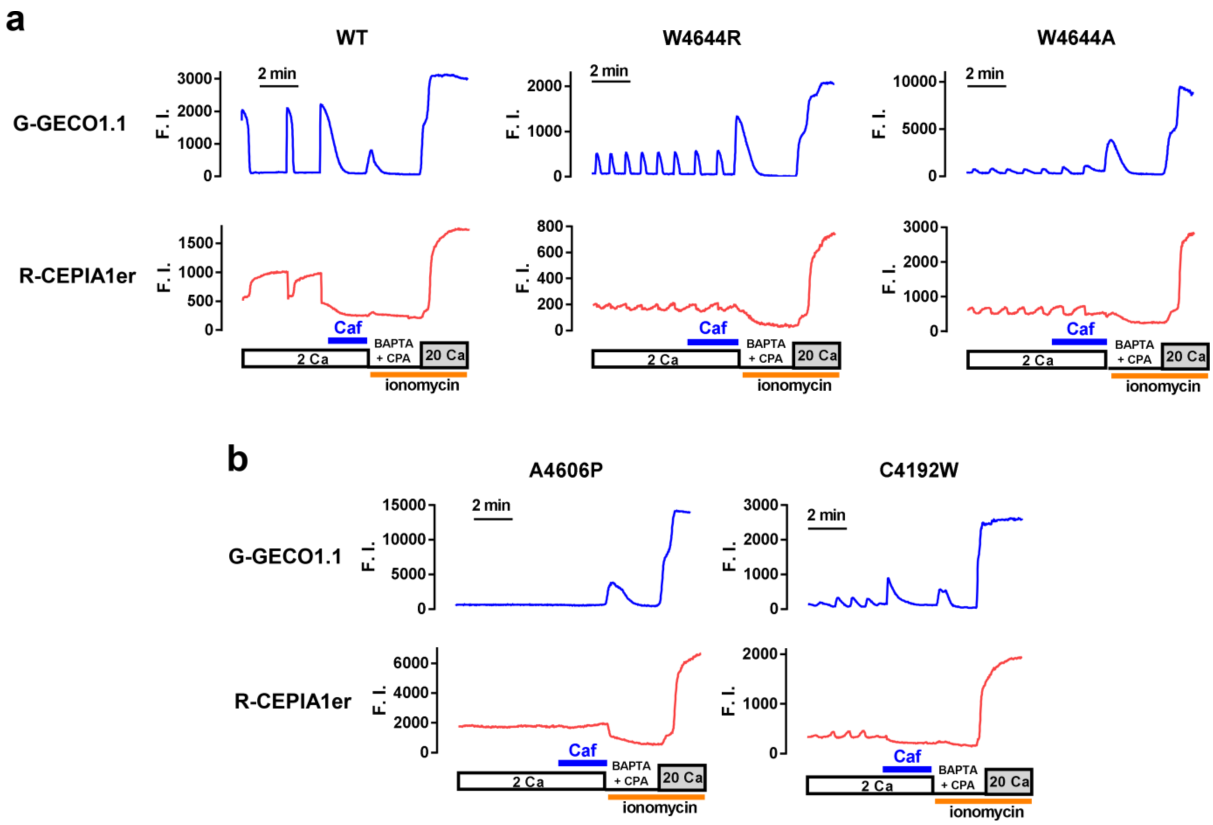

Supplementary Figure 2. Raw fluorescence intensity data of G-GECO1.1 and R-CEPIA1er signals in cells expressing WT and mutant RyR2. (a) WT (*left*), W4644R (*centre*) and W4644A (*right*) shown in Fig. 2a-c. (b) A4606P (*left*) and C4192W (*right*) shown in Fig. 7d. After washout of caffeine (*Caf*), Ca^{2+} free Krebs solution containing 20 μM ionomycin, 5mM BAPTA and 20 μM cyclopiazonic acid (CPA) were applied to obtain minimal fluorescence intensity (F_{min}). Finally, F_{max} was obtained with Krebs solution containing 20 μM ionomycin and 20 mM $CaCl_2$. Note that a transient increase of G-GECO1.1 signals at BAPTA+CPA indicates Ca^{2+} leak from the ER by ionomycin.

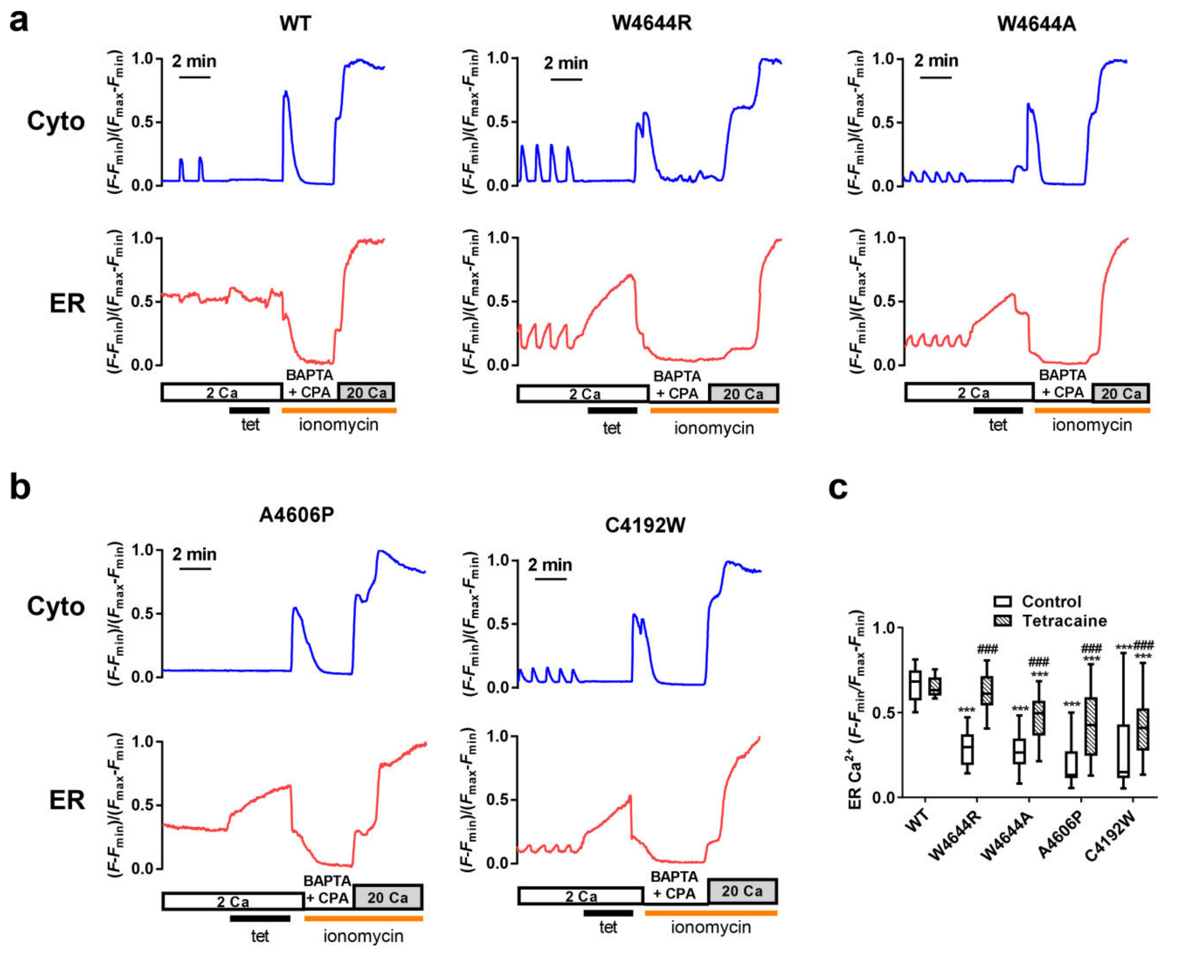

Supplementary Figure 3. Effects of tetracaine on $[Ca^{2+}]_i$ and $[Ca^{2+}]_{ER}$ signals in cells expressing WT and mutant RyR2. (a) Representative traces of WT (*left*), W4644R (*centre*) and W4644A (*right*). (b) Representative traces of A4606P (*left*) and C4192W (*right*). Fluorescent signals are expressed as $(F\text{-}F_{min})/(F_{max}\text{-}F_{min})$. Tetracaine (1 mM, *tet*) was applied at the time indicated by the black bar. Note that tetracaine effectively suppressed Ca^{2+} oscillations and greatly increased $[Ca^{2+}]_{ER}$ of the mutant cells. (c) $[Ca^{2+}]_{ER}$ of WT and mutant RyR2 cells in control (*open columns*) and with 1 mM tetracaine (*hatched columns*). Data are given as box and whisker plots displaying the minimum, first quartile, median, third quartile, and maximum values (n = 17–49). *** indicates statistical significance with $p < 0.0001$ vs WT using one-way ANOVA followed by Dunnett's post hoc test. ### indicates $p < 0.0001$ vs control using unpaired two-tailed Student's t-test.

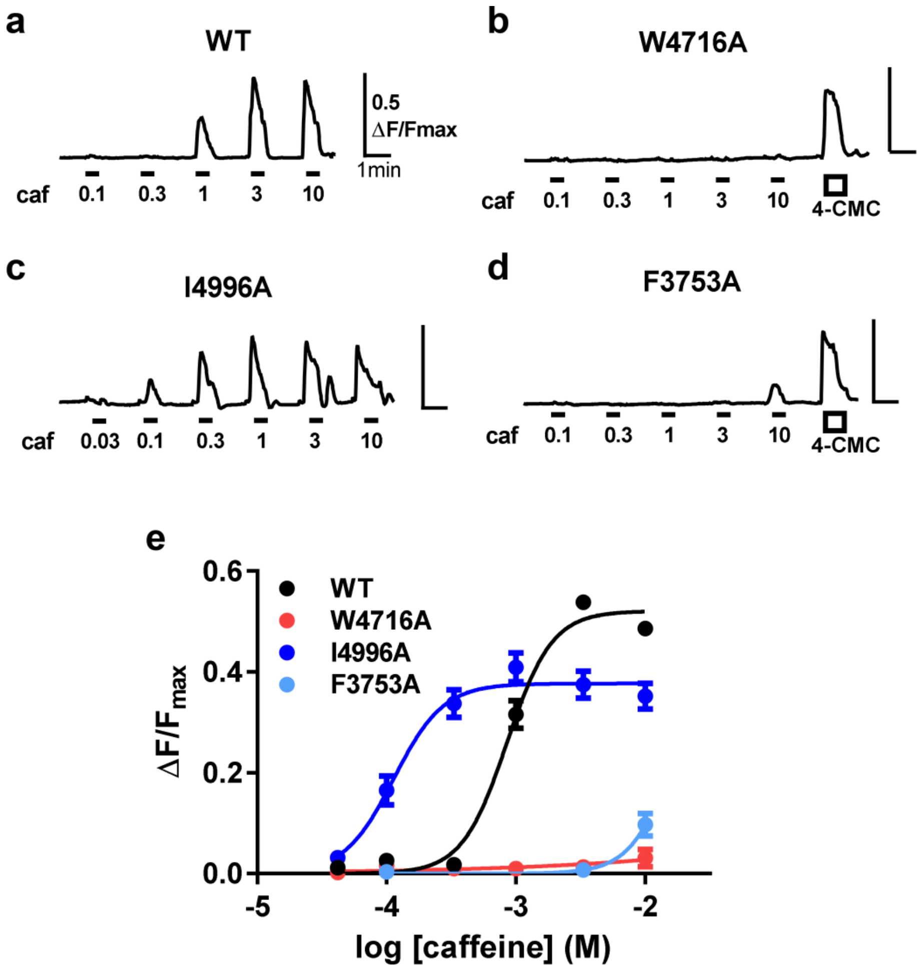

Supplementary Figure 4. Effects of alanine substitution of residues in the putative caffeine binding site in RyR1. (a-d) Representative traces of fluo-4 signals for HEK293 cells expressing WT (a), W4716A (b), I4996A (c), and F3753A (d) by an application of caffeine. Cells were loaded with fluo-4 AM and response to caffeine treatments were recorded. The *short horizontal bars* indicate the application of caffeine (0.03-10 mM) or 100 μM 4-chloro-m-cresol (4-CmC). The fluorescence signal (F) was normalized to the maximal fluorescence intensity of fluo-4 (F_{max}) that was obtained with Krebs solution containing 20 mM $CaCl_2$ and 20 μM ionomycin at the end of each experiment. (e) Dose response relations between peak $\Delta F/F_{max}$ and caffeine concentration for WT (*black*, n = 140), W4716A (*red*, n = 70), I4996A (*blue*, n = 70) and F3753A (*light blue*, n = 95). Data are given as mean ± SEM.

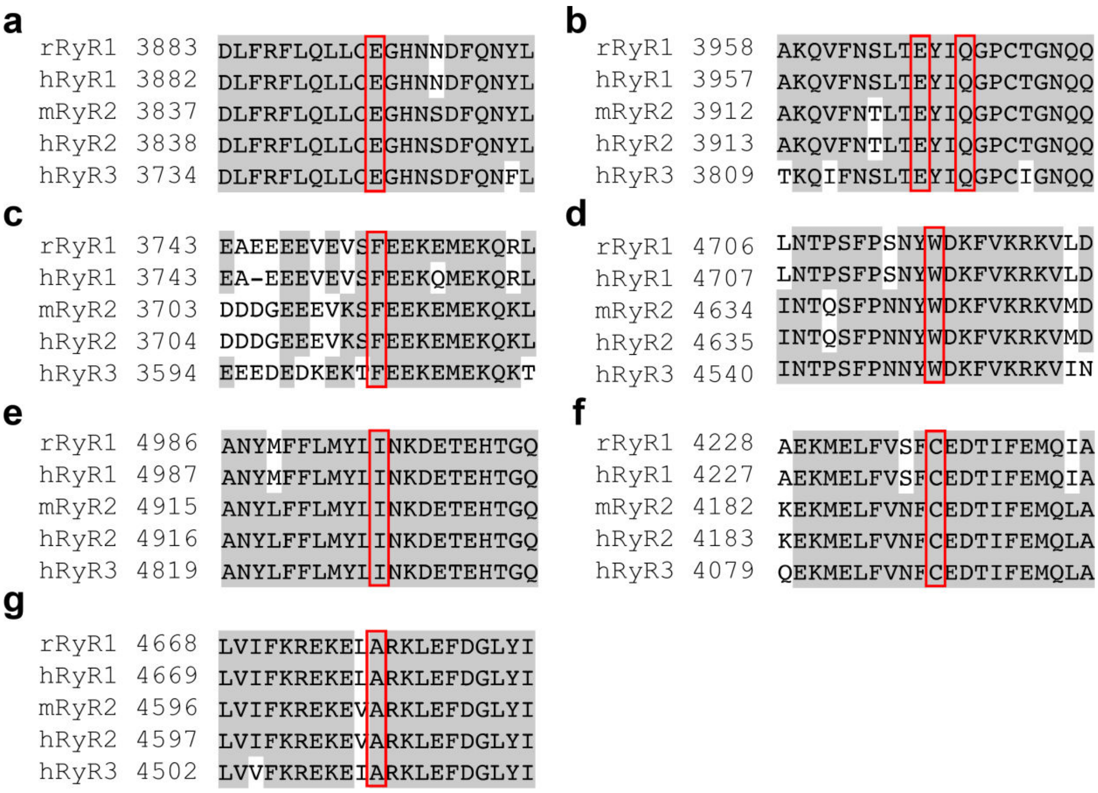

Supplementary Figure 5. Amino acid sequence alignments of the putative Ca^{2+}- and caffeine-binding and disease-associated mutant sites. Portions of sequence alignments of rabbit RyR1 (Uniprot accession number P11716) with human RyR1 (P21817), mouse RyR2 (E9Q401), human RyR2 (Q92736) and human RyR3 (Q15413) around residues important for Ca^{2+}- and caffeine-binding sites (*red boxes*); E3847 (a), E3921 and Q3924 (b), F3713 (c), W4716 (d), I4996 (e), C4238 (f) and A4678 (g). The alignment is shaded by conservation with a cut-off of 60%.

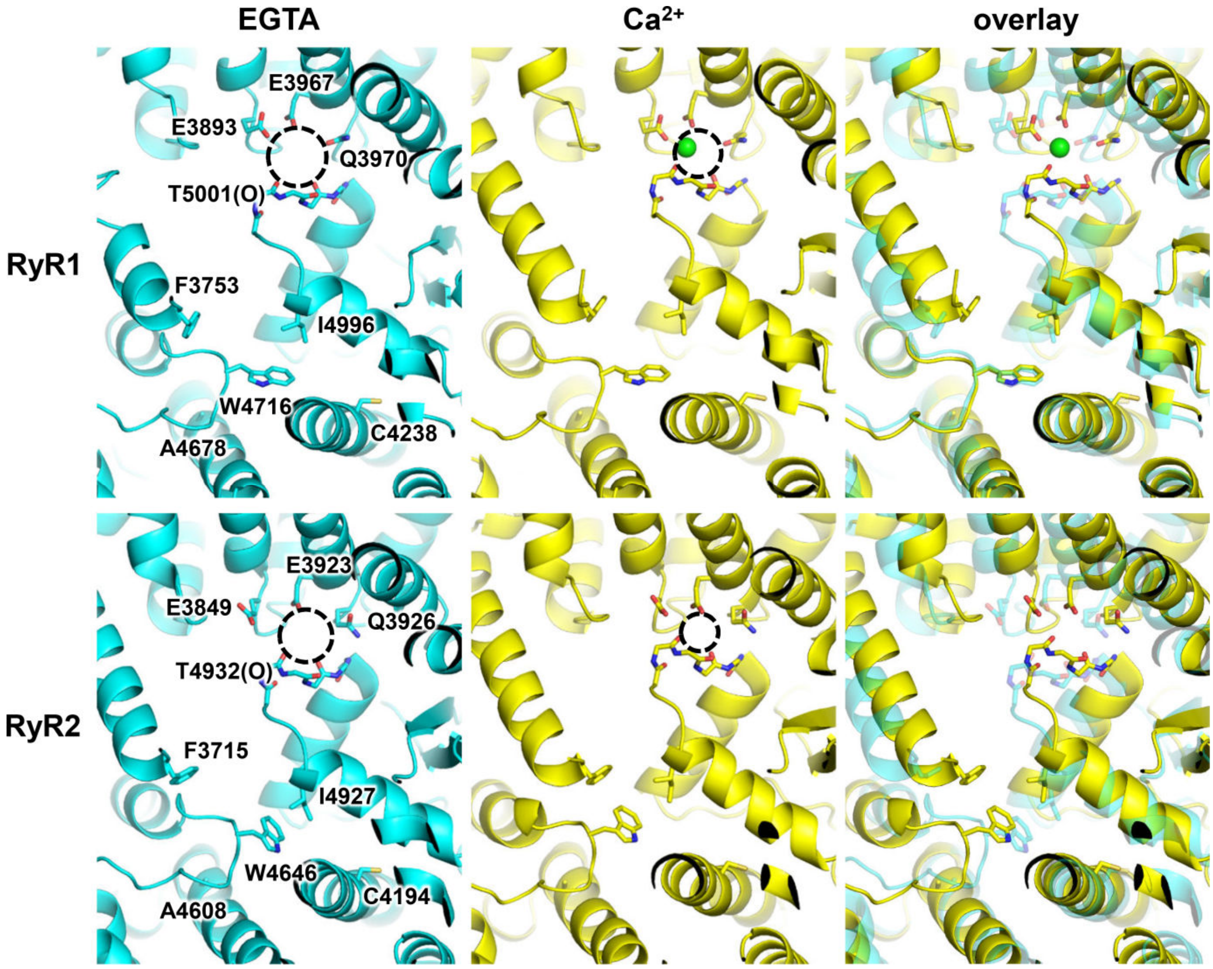

Supplementary Figure 6. Conformational changes of RyR1 and RyR2 by Ca^{2+}. The architecture of putative Ca^{2+}- and caffeine-binding sites of rabbit RyR1 (*top*) and pig RyR2 (*bottom*) in the presence of EGTA (*left*, PDB accession number 5TB0 for RyR1 and 5GO9 for RyR2) and Ca^{2+} (*centre*, 5T15 for RyR1 and 5GOA for RyR2) are depicted in ribbon representation. The two structures are overlaid in the *right* panel. The likely interacting residues of the putative Ca^{2+}- and caffeine-binding sites of RyR1 and RyR2 are labelled and the Ca^{2+}-binding pocket is indicated by a dotted circle.

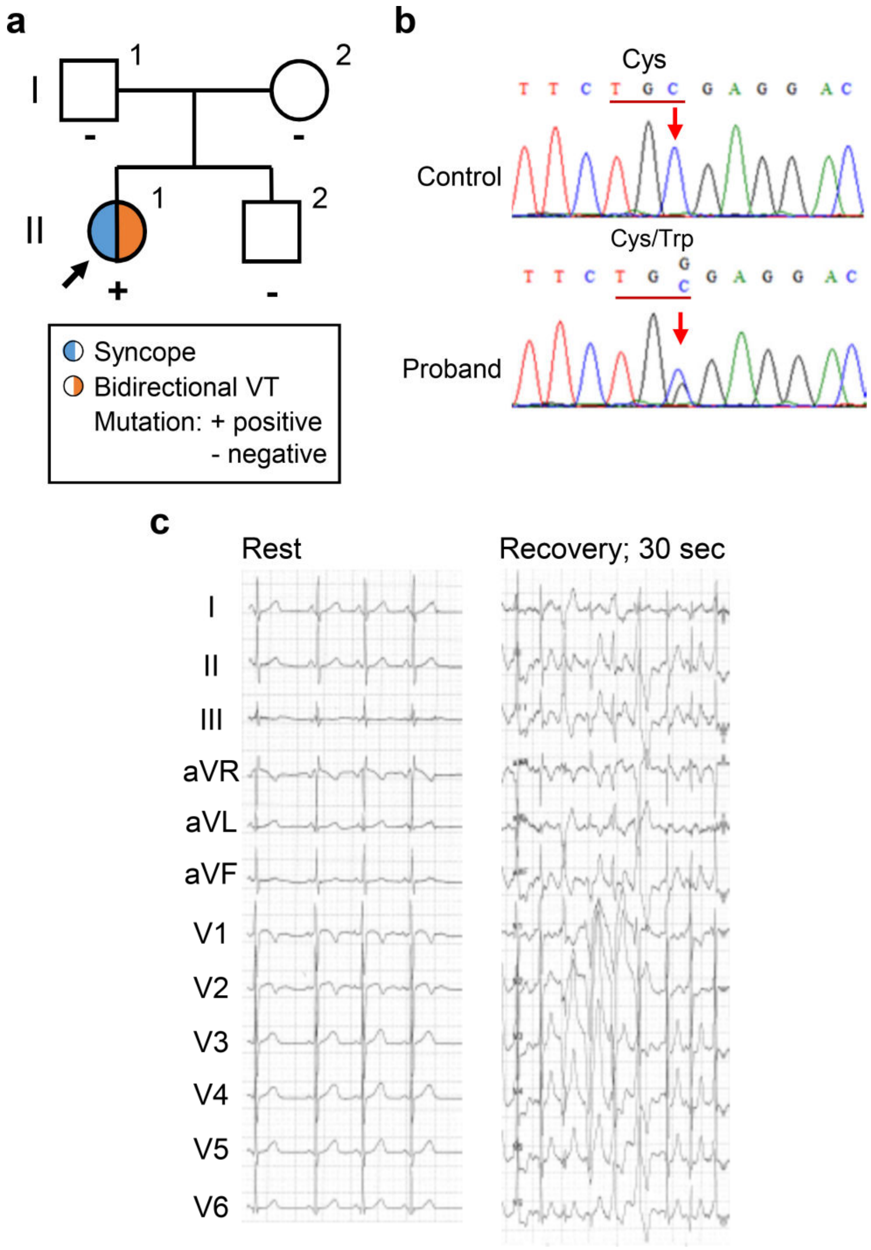

Supplementary Figure 7. Clinical information of human RyR2-C4193W carrier. (a) Family tree of the RyR2-C4193W carrier. The proband (II-1) suffered from syncope, and bidirectional VT was recorded in the exercise stress test. RyR2-C4193W was identified in the genetic analysis. No other family members showed symptoms and carried the mutation. (b) Sequence electrophoresis. Cytosine at 12579 in RyR2 was substituted to guanine leading to the substitution of cysteine (*Cys*) at codon 4193 to tryptophan (*Trp*) in the proband. (c) Exercise stress test of the proband. *Left*, ECG recorded before exercise stress test shows no abnormal finding. *Right*, ECG recorded just after the exercise stress test shows bidirectional VT.

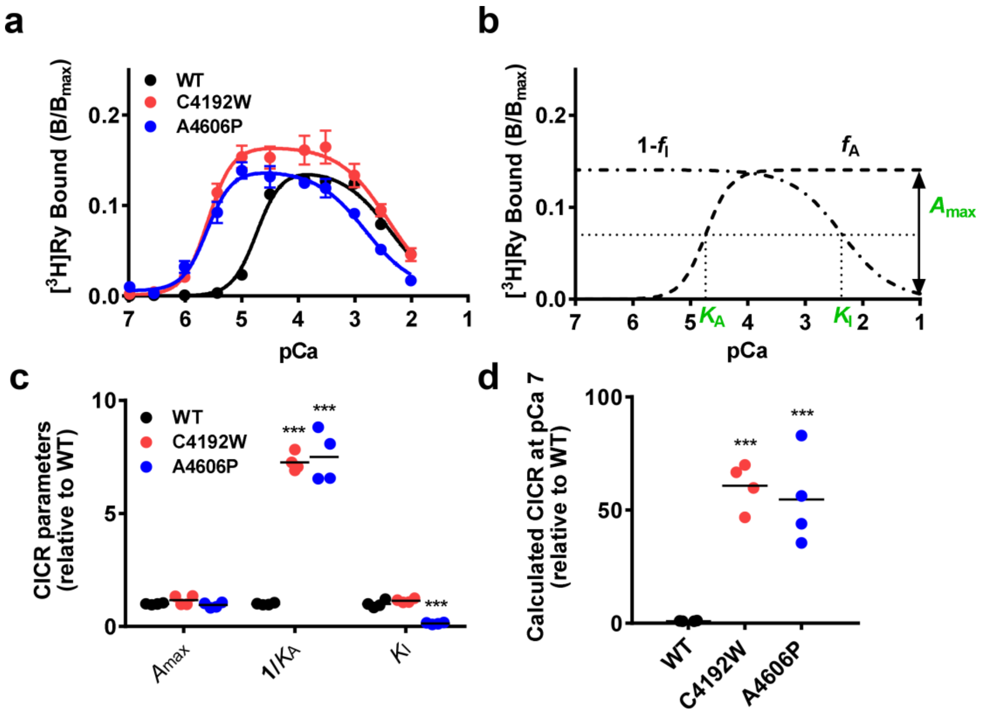

Supplementary Figure 8. Calculation of the channel activity of disease-associated RyR2 mutants at resting Ca^{2+} using three parameters of CICR. (a) Ca^{2+}-dependent [^{3}H]ryanodine binding of WT (*black*), C4192W (*red*) and A4606P (*blue*) RyR2. (b) Ca^{2+}-dependent channel activity is determined by fraction of A-site occupied by Ca^{2+} (f_A, *broken line*), fraction of I-site free from Ca^{2+} ($1–f_I$, *dashed line*) and the gain (A_{max}) that sets the maximal attainable activity. Three CICR parameters (K_A, K_I and A_{max}) were obtained by fitting the data to equations (1)-(3). (c) CICR parameters for WT, C4192W and A4606P. $1/K_A$ was increased 8-fold in both mutants compared with WT RyR2. Data are given as mean and individual values (n = 4). *** indicates statistical significance with $p < 0.0001$ vs WT using one-way ANOVA followed by Dunnett's post hoc test. (d) CICR activity for WT, C4192W and A4606P at pCa 7 was calculated using the obtained CICR parameters. Data are given as mean and individual values (n = 4). Both mutants show 60-fold greater activity than WT RyR2.

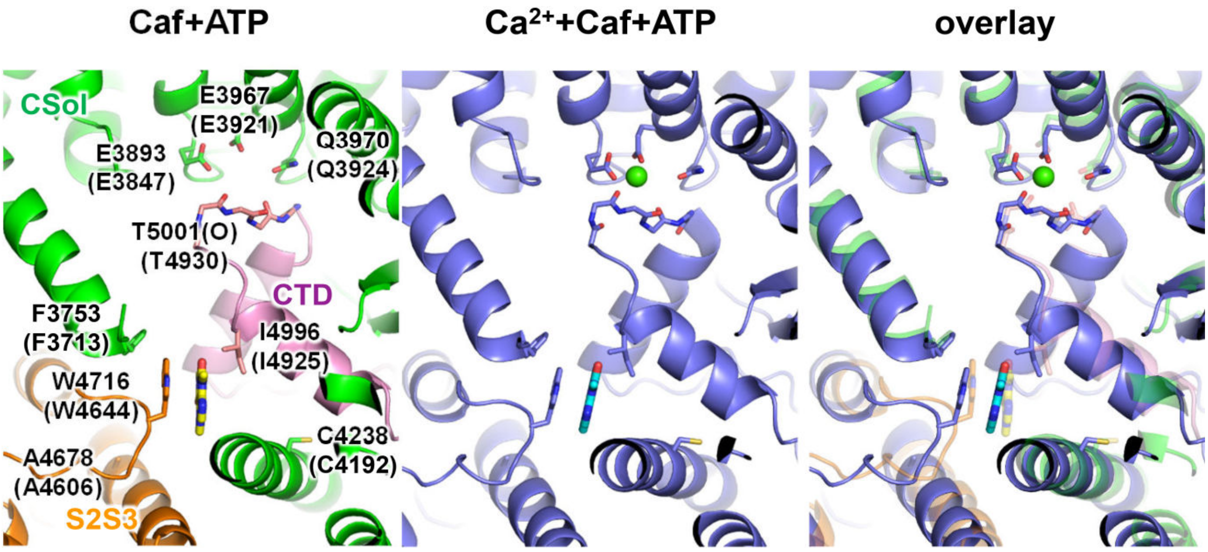

Supplementary Figure 9. Conformational change of RyR1 by caffeine and Ca^{2+} in the presence of ATP. The architecture of putative Ca^{2+}- and caffeine-binding sites of rabbit RyR1 in the presence of caffeine and ATP (*left*) (PDB accession number 5TAP) and in the presence of Ca^{2+}, caffeine and ATP (*centre*, 5T9V) are depicted in ribbon representation. The two structures are overlaid in the *right* panel. The likely interacting residues of the putative Ca^{2+}- and caffeine-binding sites are labelled.

Supplementary Table 1. Amino acid residues in mammalian RyR homologs corresponding to the putative Ca^{2+}- and caffeine-binding sites and disease-associated mutation sites. Uniprot accession numbers are indicated below the protein name. The originally identified residues are underlined.

RyR1 (rabbit) P11716	RyR1 (human) P21817	RyR2 (mouse) E9Q401	RyR2 (human) Q92736	RyR3 (human) Q15413
		Ca^{2+}-binding site		
E3893	E3892	E3847	E3848	E3744
E3967	E3966	E3921	E3922	E3818
Q3970	E3969	Q3924	Q3925	Q3821
		Caffeine-binding site		
F3753	F3752	F3713	F3714	F3524
W4716	W4717	W4644	W4645	W4550
I4996	I4997	I4925	I4926	I4829
		Disease-associated mutation sites		
C4238	C4239	C4192	C4193	C4089
A4678	A4679	A4606	A4607	A4512

Supplementary Table 2. Nucleotide sequences for PCR primers used for mutagenesis of RyR1 and RyR2. Mutated nucleotides are underlined.

Isoform	Mutation	Forward primer (5' to 3')	Reverse primer (5' to 3')
**RyR1**	**F3753A**	CTGAGGAGAAAGAGATGGAGA	CGGAGACCTCAACCTCCTCTT
**RyR1**	**W4716A**	CGGACAAGTTTGTCAAGCGGA	CGTAGTTGCTGGGGAAAGACG
**RyR1**	**I4996A**	CAAACAAGGACGAGACGGAGC	CCAGATACATCAAGAAGAACA
**RyR2**	**W4644R**	CGGGATAAATTTGTGAAAAGA	GTAGTTATTGGGAAATGACTG
**RyR2**	**W4644A**	CGGATAAATTTGTGAAAAGAA	CGTAGTTATTGGGAAATGACT
**RyR2**	**W4644I**	TCGATAAATTTGTGAAAAGAAA	TGTAGTTATTGGGAAATGACT
**RyR2**	**W4644L**	TGGATAAATTTGTGAAAAGAA	GGTAGTTATTGGGAAATGACT
**RyR2**	**I4925A**	CAAACAAGGATGAAACAGAAC	CGAGATACATCAGAAAAAACA
**RyR2**	**F3713A**	CTGAAGAAAAGGAAATGGAAA	CGCTCTTCACTTCTTCTTCAC
**RyR2**	**E3847A**	CGGGGCACAACTCAGACTTTC	CACAAAGCAGCTGCAGGAATC
**RyR2**	**E3847D**	CGGGCACAACTCAGACTTTCA	TCACAAAGCAGCTGCAGGAAT
**RyR2**	**E3921A**	CGTACATCCAGGGCCCTTGTA	CCGTGAGCGTGTTGAACACCT
**RyR2**	**E3921D**	CTACATCCAGGGCCCTTGTAC	TCCGTGAGCGTGTTGAACACC
**RyR2**	**Q3924A**	CGGGCCCTTGTACTGGAAATC	CGATGTACTCCGTGAGCGTGT
**RyR2**	**Q3924E**	GAGGGCCCTTGTACTGGAAAT	GATGTACTCCGTGAGCGTGTT
**RyR2**	**C4192W**	GGAGGACACCATCTTTGAGAT	CAGAAGTTCACAAAGAGCTCC
**RyR2**	**A4606P**	CCACGGAAGTTGGAGTTTGAC	CACTTCCTTCTCTCGCTTAAA
